# Hexokinase-3 upregulation and histone H3K18 lactylation mediate murine liver fibrosis induced by *Echinococcus multilocularis*

**DOI:** 10.1371/journal.pntd.0014582

**Published:** 2026-07-31

**Authors:** Yurun Miao, Lixian Shan, Jing Liu, Wei Cui, Xiangyang Yang, Lei Xiu, Wenbin Yang, Chunfu Li, Wenhui Liu, Yan Wang, Ting Zhang, Wei Hu

**Affiliations:** 1 The State Key Laboratory of Reproductive Regulation and Breeding of Grassland Livestock, School of Life Sciences, Inner Mongolia University, Hohhot, China; 2 Department of Medicine, College of Hetao, Bayannur, China; 3 National Health Commission Key Laboratory of Echinococcosis Prevention and Control, Tibet Autonomous Region Center for Disease Control and Prevention, Lhasa, Tibet Autonomous Region, China; 4 National Institute of Parasitic Diseases, Chinese Center for Disease Control and Prevention (Chinese Center for Tropical Diseases Research); National Key Laboratory of Intelligent Tracking and Forecasting for Infectious Diseases, NHC Key Laboratory of Parasite and Vector Biology, WHO Collaborating Centre for Tropical Diseases, Shanghai, China; 5 State Key Laboratory of Genetic Engineering, Ministry of Education Key Laboratory of Contemporary Anthropology, Collaborative Innovation Center for Genetics and Development, School of Life Sciences, Fudan University, Shanghai, China; University of Liverpool, UNITED KINGDOM OF GREAT BRITAIN AND NORTHERN IRELAND

## Abstract

Alveolar echinococcosis (AE), caused by *Echinococcus multilocularis* infection, leads to destructive hepatic lesions and severe hepatic fibrosis. Lactylation, a post-translational modification, has been linked to fibrotic progression in hepatocytes. However, the role of *E. multilocularis* infection in hepatic lactylation remains unclear. This study aims to elucidate the relationship between hepatic fibrosis and lactylation induced by *E. multilocularis* infection and fill this knowledge gap. The results showed that lactate concentrations in the livers and sera of AE mice were significantly elevated compared with those in the control group. The relative mRNA expression of hexokinase isoforms in the liver tissue of AE mouse models revealed significant upregulation of hexokinase-3 (HK3), a key enzyme regulating lactate production. Immunofluorescence staining revealed substantial colocalization between HK3 and TGF-β1 in hepatic lesions of AE mice. Western blot analysis using a pan-lactylation antibody showed significantly elevated lactylation levels in the livers of AE mice compared with controls, with a significant increase in histone H3 lysine 18 lactylation (H3K18la). CUT&Tag analysis revealed differential enrichment of H3K18la at multiple fibrosis-associated genes in AE mouse liver. In *vitro*, AML-12 hepatocytes exposed to *E. multilocularis* culture supernatant showed significant upregulation of TGF-β1, HK3, and histone lactylation. HK3 knockdown significantly reduced the induction of H3K18la and fibrosis-related proteins, whereas exogenous lactate supplementation significantly increased these responses despite HK3 silencing, confirming that lactate acts downstream of HK3 to drive histone lactylation and fibrogenesis. Corosolic acid, a compound that limits HK3-mediated lactate production, attenuated histone lactylation and fibrosis-related protein expression. These findings indicate that HK3-driven histone lactylation at H3K18 contributes to hepatic fibrosis in AE, and suggest that HK3 may serve as promising therapeutic targets for AE.

## Author summary

Alveolar echinococcosis (AE), caused by *Echinococcus multilocularis* infection, leads to severe hepatic fibrosis, but the underlying molecular mechanisms remain unclear, hindering the development of effective therapies. Using a mouse model, we demonstrated that *E. multilocularis* infection significantly increases lactate levels in liver tissue and blood, while upregulating hexokinase-3 (HK3), a key enzyme in lactate production. We further discovered that infected liver cells accumulated more “lactylation” tags on their DNA-packaging proteins, specifically at the H3K18 site. This molecular change was associated with altered activity of several fibrosis-related genes that may contribute to liver scar tissue formation. By silencing HK3 in cells and then adding lactate back, we confirmed that the lactate produced by HK3 drives these lactylation changes and fibrosis. Notably, corosolic acid, which reduces HK3-driven lactate production, suppressed key steps of this disease process. Our findings reveal that HK3-mediated lactate production and subsequent histone lactylation promote AE-induced hepatic fibrosis, highlighting HK3 as potential therapeutic targets. This work fills a critical knowledge gap in AE pathogenesis, identifies a promising treatment target, and may provide insights for investigating similar metabolic-epigenetic mechanisms in other neglected tropical diseases.

## Introduction

Alveolar echinococcosis (AE) is a zoonotic parasitic disease caused by infection with the larval stage of *Echinococcus multilocularis*. This pathogen primarily inhabits the cold and temperate regions of the Northern Hemisphere, with a particularly high prevalence in China’s Qinghai-Tibetan Plateau [1]. Globally, AE accounts for approximately 18,200 new infections annually and is responsible for an estimated loss of 6.66 million disability-adjusted life years (DALYs) [2]. AE is considered one of the most severe emerging and re-emerging parasitic diseases threatening human health due to its high mortality rate (94%) [3] and frequent post-treatment recurrence (34%) [4]. The absence of effective vaccines or highly efficient therapies further exacerbates the clinical challenge. Therefore, there is an urgent need to develop novel and effective therapeutic strategies to improve clinical outcomes for patients with AE.

The metacestodes of *E. multilocularis* infect both humans and small rodents, with the liver being the primary target organ [5] The parasite proliferates through budding and invasive infiltration, producing vesicles that penetrate deep into tissues and form irregular structures with indistinct boundaries from the surrounding hepatic parenchyma. In human AE, granulomatous inflammation develops around these parasitic vesicles, leading to severe hepatic fibrosis and necrosis, which, if left untreated, can progress to organ failure and death. During chronic *E. multilocularis* infection, host hepatocytes secrete multiple pro-fibrotic factors such as transforming growth factor β1 (TGF-β1) [6], fibroblast growth factor (FGF) [7], inducible nitric oxide synthase (iNOS) [8], nuclear factor κB (NF-κB) [9], and interleukin-10 (IL-10) [10]. These factors activate hepatic stellate cells (HSCs), induce significant metabolic alterations, and drive the development of hepatic fibrosis. Recent research indicates that HSC activation is a key driver of liver fibrogenesis [11]. However, the precise molecular mechanisms regulating HSC activation and fibrosis progression in AE remain incompletely understood, representing a major obstacle to the development of targeted antifibrotic therapies.

Lactylation is a recently discovered type of epigenetic modification, in which lactyl groups are enzymatically added to lysine residues on histone and nonhistone proteins, thereby influencing gene expression. This modification, first identified by Zhang et al. in 2019 during studies of intracellular lactate accumulation in bacterial infection [12], has since been observed in various pathological contexts and is closely associated with processes such as tumorigenesis, disease progression, immune evasion, metabolic reprogramming, and attenuation of innate immune responses [13]. Importantly, recent studies have demonstrated that histone lactylation can activate HSCs, induces their pro-fibrotic phenotype, and promotes liver fibrosis [14,15].

These findings suggest that inhibition of lactylation-mediated HSC activation could represent a promising therapeutic approach for treating liver fibrosis. However, the role of histone lactylation in AE-associated liver fibrogenesis remains elusive.

In this study, abnormal lactate accumulation was identified in the livers and peripheral blood of *E. multilocularis*-infected mice, which was associated with increased HK3 expression. Both HK3 and TGF-β were highly co-localized in the livers of infected mice, indicating a strong association between lactate accumulation and hepatic fibrosis in hepatic alveolar echinococcosis (HAE). Histone H3K18 lactylation (H3K18la) was identified as a key molecular event in this process. Moreover, *in vitro* experiments revealed that corosolic acid inhibited the upregulation of HK3 and the expression of fibrosis-related proteins induced by *E. multilocularis* infection. These findings indicate that hepatic histone lactylation, particularly H3K18la, is associated with AE-induced fibrogenesis and suggest that targeting HK3 may offer therapeutic potential for AE.

## Materials and methods

### Ethics statement

All procedures involving animals were approved by the Animal Ethics Committee of the Chinese Center for Parasitic Diseases Prevention and Control (Approval Code: IPD-2021–30) and the Animal Care and Use Committee of Inner Mongolia University (Permit No. SCXK (Inner Mongolia) 2020–0006). All measures were taken to minimize discomfort during handling

### Isolation of *Echinococcus* protoscoleces

Mongolian jirds (*Meriones unguiculatus*) were infected with protoscoleces (PSCs) of *E. multilocularis* (Qinghai isolate, China) and maintained for six months post-infection. Animals were euthanized by cervical dislocation according to institutional guidelines, and metacestode tissues were collected aseptically from the peritoneal cavity. The harvested material was rinsed with sterile phosphate-buffered saline (PBS, pH 7.4) following established procedures [16,17].

After carefully removing surrounding host tissue with sterile forceps, the metacestode masses were cut into 1–2 mm pieces using a sterile scalpel to maximize PSC release. The fragments were passed through sterile 100-mesh and 40-mesh sieves (Biosharp, Beijing, China; BS-100-CS and BS-40-CS), and the resulting suspension was transferred into centrifuge tubes. PSCs were washed five times with PBS containing 100 U/mL penicillin and 100 µg/mL streptomycin (Gibco, Grand Island, NY, USA). After each wash, tubes were allowed to stand for 5 min at room temperature to allow natural sedimentation, and the supernatant was aspirated gently. The cleaned PSCs were used for the subsequent *in vivo* and *in vitro* experiments.

### Culture of *Echinococcus* Protoscoleces *In Vitro*

The cleaned PSCs pellet was resuspended in Dulbecco’s Modified Eagle Medium (DMEM; Gibco, Auckland, New Zealand) supplemented with 10% (v/v) fetal bovine serum, 100 U/mL penicillin, and 100 µg/mL streptomycin. Cultures were maintained at 37°C in a humidified atmosphere with 5% CO_2_, and the medium was refreshed every 48 h. PSC viability and larval vesicle formation were monitored weekly using an Olympus IX71 inverted microscope (Tokyo, Japan) at ×100 magnification. PSCs showing normal motility and intact morphology were selected for infection of C57BL/6 mice and for *in vitro* induction of AML-12 cells.

### Collection of PSC culture supernatant and stimulation of AML-12 cells

PSCs were cultured in DMEM supplemented with 10% FBS for 72 h. The culture supernatant was then collected, centrifuged at 1,000 × *g* for 10 min to remove cellular debris, and filtered through a 0.22 μm syringe filter. The resulting sterile supernatant was stored at −80°C until use. To determine the optimal stimulation conditions, a CCK-8 assay was performed to screen various dilution ratios (1:1, 1:2.5, 1:5, and 1:10) and time points (0, 6, 12, 24, and 48 h), ensuring that the selected conditions effectively activated the fibrotic response without inducing significant cytotoxicity. Based on these results, the supernatant was diluted 1:2.5 (v/v) in complete DMEM and applied to cells AML-12 cells for 72 h at 37°C in 5% CO_2_.

### Animal model and parasite infection

Eight-week-old female C57BL/6 mice (20–22 g) were obtained from Beijing Sibeifu Biotechnology Co., Ltd. (Beijing, China). Animals were maintained in specific pathogen-free (SPF) conditions with unrestricted access to standard chow and water. Twenty mice were randomly divided into four groups: Group 1: Uninfected control group; Group 2: Uninfected + corosolic acid group; Group 3: *E. multilocularis*-infected group; and Group 4: *E. multilocularis*-infected + corosolic acid group.

Mice in Groups 3 and 4 were infected with 1,500 cleaned PSCs *via in situ* intrahepatic surgical implantation, which closely mimics natural hepatic infection. Mice in Group 1 underwent sham surgery without PSC implantation. Beginning at 60 dpi, Groups 2 and 4 received intraperitoneal injections of corosolic acid (CorA), a pentacyclic triterpenoid reported to modulate hexokinase activity and glucose metabolism [18], at 10 mg/kg every 3 days for a total of 5 doses. All mice were kept under identical environmental conditions (22 ± 1°C, 12 h light/dark cycle), and the infection procedures were identical across groups to ensure consistency.

### Blood lactate and hepatic histopathological analysis in mice

At 12 weeks post-infection, C57BL/6 mice were anesthetized with isoflurane (induction 3–5%; maintenance 1–2% in oxygen). Blood samples were collected by orbital enucleation, transferred to serum separation tubes, and centrifuged at 3,000 rpm for 15 min at 4°C. Serum lactate levels were quantified using an automated biochemical analyzer (BS-240VET; Mindray, Shenzhen, China) following the manufacturer’s instructions.

After blood collection, mice were euthanized by cervical dislocation. Liver tissues from Groups 1, 3, and 4 were fixed in 4% paraformaldehyde (Biosharp, Beijing, China) for 24 h at room temperature, embedded in paraffin, and sectioned at 3.0-5.0 µm. Histopathological changes were evaluated using hematoxylin-eosin (H&E) staining according to standard protocols [19], and hepatic fibrosis was assessed using Sirius Red and Masson’s trichrome staining as previously described [20]. H&E staining involved hematoxylin staining for 5 min (preceded by 1 min pretreatment with a high-concentration staining solution) and eosin staining for 15 s, with intermediate rinsing under running tap water for 30 min and subsequent dehydration via 95% ethanol and gradient ethanol before mounting in neutral balsam. For Sirius Red staining, sections were incubated in Sirius Red A solution at 65°C for 30 min, sequentially treated with modified Sirius Red B solution (2 min) and C solution (30 min), briefly rinsed with distilled water, then rapidly dehydrated in three anhydrous ethanol baths (5 s each), cleared in xylene for 5 min, and mounted with neutral balsam. Masson’s trichrome staining included overnight incubation in Masson A solution, 1 min staining with a 1:1 mixture of Masson B and C solutions, 6 min incubation in Masson D solution, 1 min staining in Masson E solution, 30 s treatment in Masson F solution (without intermediate washing), differentiation with 1% acetic acid, followed by dehydration through anhydrous ethanol, xylene clearing, and neutral balsam mounting.

### RT-qPCR Analysis of TGF-β, α-SMA, and Hexokinase Isoenzymes Expression in AML-12 Cells and Mice Livers

To determine whether *E. multilocularis*-induced hepatic fibrosis is associated with lactate accumulation, mRNA expression levels of pro-fibrotic markers and members of the hexokinase family were measured in AML-12 cells and mouse liver tissues by reverse transcription quantitative PCR (RT-qPCR).

Total RNA from liver samples or AML-12 cells was isolated using TRIzol reagent (Invitrogen, Carlsbad, CA, USA) with the procedure undertaken in strict accordance with the manufacturer’s protocol [20]. RNA concentration and purity were assessed with a NanoDrop 2000 spectrophotometer (Thermo Fisher Scientific, Waltham, MA, USA). First-strand cDNA was synthesized from 1 μg of total RNA using the PrimeScript RT reagent kit with gDNA Eraser (Takara Bio, Otsu, Japan). The 20 μL reaction contained 1.0 μL PrimeScript RT Enzyme Mix I, 1.0 μL RT Primer Mix, 4.0 μL 5 × PrimeScript Buffer, and RNase-free ddH_2_O to volume. Reverse transcription was carried out at 37°C for 15 min, followed by 5 s at 85°C.

RT-qPCR reactions were performed using TB Green Premix Ex Taq II (Takara Bio) on a StepOnePlus Real-Time PCR System (Thermo Fisher Scientific). Each 20 μL reaction contained 10 μL TB Green Premix, 0.4 μL each of forward and reverse primers (10 μM), 2 μL cDNA template, and 7.2 μL nuclease-free water. Thermal cycling involved an initial denaturation at 95°C for 30 s, followed by 40 cycles at 95°C for 5 s and annealing/extension at 60°C for 30 s. A melting curve was generated to verify amplification specificity.

The target genes included two key markers of hepatic fibrosis, transforming growth factor-β1 (*Tgfb1*), and alpha-smooth muscle actin (*Acta2*), as well as four hexokinase isoenzymes: hexokinase 1 (*Hk1*), hexokinase 2 (*Hk2*), hexokinase 3 (*Hk3*), and hexokinase 4 (*Hk4*). Glyceraldehyde-3-phosphate dehydrogenase (*Gapdh*), the housekeeping gene, served as the internal control. Relative expression levels were calculated using the 2^−ΔΔCt^ method [21]. All assays were run in triplicate. Primer sequences, designed using GenBank references, are listed in S1 Table.

### Western blot analysis of fibrosis-related proteins and histone lactylation in AML-12 cells and mice livers

Western blot (WB) was performed to determine whether *E. multilocularis*-induced lactate accumulation alters the expression of fibrosis-related proteins and histone lactylation in AML-12 cells and liver tissues. The procedure for WB followed previously described methods [22,23]. Uncropped blots are shown in S1 Raw Gel.

Liver tissues or cultured cells were homogenized in RIPA lysis buffer (P0013B, beyotime, China) supplemented with protease and phosphatase inhibitors (78443, Thermo Fisher Scientific, USA); the lysates were centrifuged at 13,000 × *g* for 30 min at 4°C to collect cytoplasmic fractions, while nuclear proteins were isolated per standard protocols. Protein concentrations were determined using the BCA assay kit (Thermo Fisher Scientific, USA). Equal amounts of protein were mixed with sample buffer, denatured at 95°C for 10 min, separated by SDS–PAGE, and transferred onto PVDF membranes. Following transfer, membranes were blocked for 1 h at room temperature in 5% BSA dissolved in 1 × Tris-buffered saline with 0.1% Tween-20 (TBS-T), then incubated overnight at 4°C with primary antibodies against α-SMA, TGF-β1, HK3, pan-L-lactyllysine, H3K18la, H3K14la, and GAPDH (S2 Table for sources and dilutions). After three 5-min washes with TBS-T, membranes were incubated for 1 h at room temperature with horseradish peroxidase-conjugated Goat Anti-Rabbit IgG H&L (1:10,000; Abcam, ab6721), followed by three additional TBS-T washes to remove excess antibody. Protein bands were visualized using the SuperSignal West Pico PLUS ECL chemiluminescent reagent (Thermo Fisher Scientific) and imaged with a Bio-Rad gel documentation system.

### Immunofluorescence staining

Cellular immunofluorescence was performed as previously described [24]. AML-12 cells were seeded onto 12-well chamber slides and fixed in 4% paraformaldehyde for 15 min. Cells were permeabilized with 0.1% Triton X-100 for 10 min and blocked at room temperature for 30 min with 5% BSA prepared in 1 × PBST. Slides were then incubated overnight at 4°C with primary antibody against HK3 (1:200; Abclonal, A8428). After three rounds of washes with PBST, cells were incubated for 1 h at room temperature in the dark with Goat anti-Rabbit IgG (H + L) Highly Cross-Adsorbed Secondary Antibody (1:500; Thermo Fisher Scientific, A32731). Nuclear staining was performed with 4,’6-diamidino-2-phenylindole (DAPI) for 10 min. After three additional rounds of washing with PBST, an anti-fade mounting medium was applied. Images were acquired using a fluorescence or confocal laser scanning microscope.

Tissue immunofluorescence was conducted using previously described procedures [25]. Mouse livers were snap-frozen in optimal cutting temperature (OCT) compound on dry ice and stored at −80°C. Sections were prepared at 7 μm thickness using a cryostat, baked, and rehydrated. Antigen retrieval was performed in citric acid buffer (pH 6.0), followed by permeabilization with 0.1% Triton X-100 in PBS and blocking with 5% goat serum in PBS containing 0.5% BSA for 30 min. Sections were incubated with primary antibodies against HK3 (1:200; Abclonal, A8428) and TGF-β (1:200; Abcam, ab315254), Collagen I (1:200; Servicebio, GB155707) and Collagen III (1:200; Servicebio, GB121323). After washing, slides were incubated with Goat anti-Rabbit IgG (H + L) Highly Cross-Adsorbed Secondary Antibody (1:500; Thermo Fisher Scientific, A32731). Nuclei were counterstained with DAPI for 5 min. Sections were mounted with ProLong Gold and allowed to cure overnight at room temperature. Images were obtained using an LSM800 (ZEISS; Germany) confocal microscope.

### Immunohistochemistry (IHC)

Paraffin-embedded tissue sections were deparaffinized, rehydrated, and subjected to antigen retrieval in sodium citrate buffer (10 mM, pH 6.0). Endogenous peroxidase activity was quenched with 3% H_2_O_2_, followed by blocking with 5% BSA. Sections were incubated with primary antibody against Collagen I and Collagen III (1:500) overnight at 4℃, followed by HRP-conjugated secondary antibody (1:500) for 1 h at room temperature. Signals were developed with DAB and counterstained with hematoxylin. Images were acquired under a light microscope.

### Cleavage Under Targets and Tagmentation (CUT&Tag) analysis of mice livers using anti-H3K18la antibody

#### CUT&Tag.

CUT&Tag was performed using the NovoNGS CUT&Tag 4.0 High-Sensitivity Kit (Vazyme Biotech, N259-YH01) following the manufacturer’s protocol [26]. Native nuclei were isolated from frozen liver tissues of *E. multilocularis*-infected and control mice as previously described [27]. Purified nuclei (1.5 × 10^5^) were washed twice with Wash Buffer and incubated with 10 μL of concanavalin A-coated magnetic beads for 10 min at room temperature. After removal of the unbound fraction, bead-bound nuclei were resuspended in Primary Antibody Buffer (AB) containing Anti-L-Lactyl-Histone H3 (Lys18) Rabbit mAb (PTM-1427RM; 1:50) or normal rabbit IgG (CST, 2729S) and incubated overnight at 4°C.

Following primary antibody removal using a magnetic stand, samples were incubated for 1 h at room temperature with secondary antibody (Goat anti-Rabbit IgG H&L, PTM-6252; 1:200 in AB). After 2–3 washes in AB, the pA-Tn5 adapter complex (1:80 dilution in ChiTag Buffer) was added and incubated for 1 h at room temperature. Samples were washed 2–3 times in ChiTag Buffer (5 min each), resuspended in Tagmentation Buffer, and incubated at 37°C for 1 h.

DNA was purified using Tagment DNA Extract Beads. For library amplification, 20 μL of purified DNA was mixed with 2.5 μL each of a universal i5 primer and a uniquely barcoded i7 primer, followed by the addition of 25 μL of 2 × HiFi AmpliMix. The mixture was introduced into a thermocycler with a heated lid and PCR amplification was performed under the following conditions: 72°C for 3 min; 98°C for 30 s; 13 cycles of 98°C for 15 s, 63°C for 8 s, and 72°C for 5 s; a final extension at 72°C for 2 min; and a hold at 10°C. Libraries were purified using NovoNGS DNA Clean Beads.

#### DNA sequencing.

Library quality was assessed using the Agilent 5400 system (Agilent Technologies, USA) and quantified by qPCR (1.5 nM). Sequencing was conducted on the Illumina NovaSeq X-Plus platform. Qualified libraries were pooled and sequenced using a paired-end 150 bp (PE150) strategy, depending on the library’s effective concentration and the required volume of data.

#### Quality control.

Raw sequencing reads in FASTQ format were processed with Cutadapt to remove adapter sequences, poly-N reads, and low-quality reads. High-quality clean reads were retained for downstream analyzes. Q20, Q30, and GC content were calculated from the clean data to assess data quality.

#### Reads mapping to the reference genome.

Adaptor sequences were removed from raw reads to generate clean paired-end reads. These reads were aligned to the reference mouse genome using Bowtie2 under default parameters. Alignment results were stored in BAM format for downstream analyzes.

#### Peak calling.

MACS2 was used to identify enriched regions (peaks) with the aligned BAM files as input. Peak calling was performed using a q-value cutoff of < 0.05.

### Motif analysis

Motif enrichment analysis was conducted using the findMotifsGenome.pl script in Hypergeometric Optimization of Motif EnRichment (HOMER; http://homer.ucsd.edu/homer/). Peak BED files and the corresponding genome FASTA files were used as input. HOMER extracted DNA sequences based on peak coordinates and compared them with known motif databases to identify significantly enriched transcription factor motifs.

### Peak annotation

Peak annotation was performed with the ChIPseeker package. Annotated genomic features, including promoters, exons, introns, and intergenic regions, were quantified, and distribution plots were generated in R.

### GO and KEGG enrichment analyzes

Gene Ontology (GO) enrichment analysis was performed to investigate the biological functions associated with peak-linked genes [28]. ClusterProfiler was used to identify enriched GO terms, with multiple-testing correction applied (Padj). KEGG pathway enrichment analysis was also carried out using ClusterProfiler, and pathways with q-values < 0.05 were considered significant [29].

### Differential peak analysis

Differentially enriched peaks were identified through the following steps: 1. Peak files from all samples were merged using BEDTools; 2. Read counts for the unified peak set were quantified using BEDTools multicov; 3. DESeq2 was applied to calculate differential enrichment. Peaks with |log_2_ fold change| > 1 and *P* < 0.05 were defined as significantly differential.

### Co-immunoprecipitation (Co-IP)

AML-12 cells stimulated with *E. multilocularis* protoscolex culture supernatant were lysed in RIPA buffer containing PMSF (P0100, Solarbio, China) at 4 °C for 30 min, and total protein was collected by centrifugation (12,000 × g, 30 min, 4 °C); an aliquot was retained as input. The lysate was incubated overnight at 4 °C with either an IgG isotype control or an anti-HK3 antibody, followed by Protein A/G agarose (Thermo, 20421) for 3–4 h. After washing three times, the immunocomplexes were eluted in 1 × loading buffer, boiled, and collected as the IP sample. Input and IP samples were analyzed by SDS-PAGE and WB using antibodies against HK3, H3K18la, and H3K14la to determine whether lactylated histones (H3K18la and H3K14la) co-precipitate with HK3. Antibody sources and working dilutions are listed in S2 Table.

### Transfection of small interfering RNA (siRNA)

HK3 siRNAs and non-targeting negative control siRNA (si-NC) were obtained from GenePharma Inc. (Suzhou, China). The sequences of the HK3 siRNAs were as follows (5’-3’): si-1 GGGCCAGGAUGUUGUGUAUTT; si-2, GCCCUCAGGUAGCUUAGAATT; si-3, GCAGUGUGCAGAUCAUCAATT; si-3, GCCAGGAUGUGGUCCAGUUTT. Among these, si-4 was selected for subsequent functional experiments based on its knockdown efficiency. Transient transfection was performed using Lipofectamine 3000 (Invitrogen, USA) at a final siRNA concentration of 10 μM according to the manufacturer’s instructions. Six hours after tranfection, the medium was replaced with complete medium and cells were cultured for an additional 48 h. Knockdown efficiency was confirmed by Western blot.

### CorA treatment and lactate supplementation of AML-12 cells

To evaluate the effect of CorA on *E. multilocularis-*induced fibrosis, AML-12 cells were pre-treated with 10 μM CorA for 1 h, followed by stimulation with *E. multilocularis* culture supernatant, and maintained for 72 h. To assess the role of lactate independently of HK3, HK3-knockdown AML-12 cells were treated with 10 mM lactic acid (L6402, Sigma-Aldrich) for 72 h in the absence of *E. multilocularis* culture supernatant. To further examine whether CorA acts on the lactate-driven phenotype, HK3-knockdown cells were pre-treated with 10 μM CorA for 1 h, followed by the addition of 10 mM lactate acid, and maintained for 72 h.

### Statistical analysis

Continuous variables are presented as mean ± standard deviation. Comparisons between two groups were performed using Student’s *t*-test, whereas analyzes involving three or more groups were conducted using two-way analysis of variance (ANOVA). Graphs were generated using GraphPad Prism 10.0 (Dotmatics, Inc., Boston, USA), while band intensities and all histopathological staining results were quantified and analyzed, respectively, using ImageJ/Fiji (macOS version; National Institutes of Health, Bethesda, MD, USA; https://imagej.net/Fiji). A *P* value < 0.05 was considered statistically significant.

## Results

### *E. multilocularis* infection induces hepatic fibrosis in mice and promotes lactate accumulation

**Fig 1:**
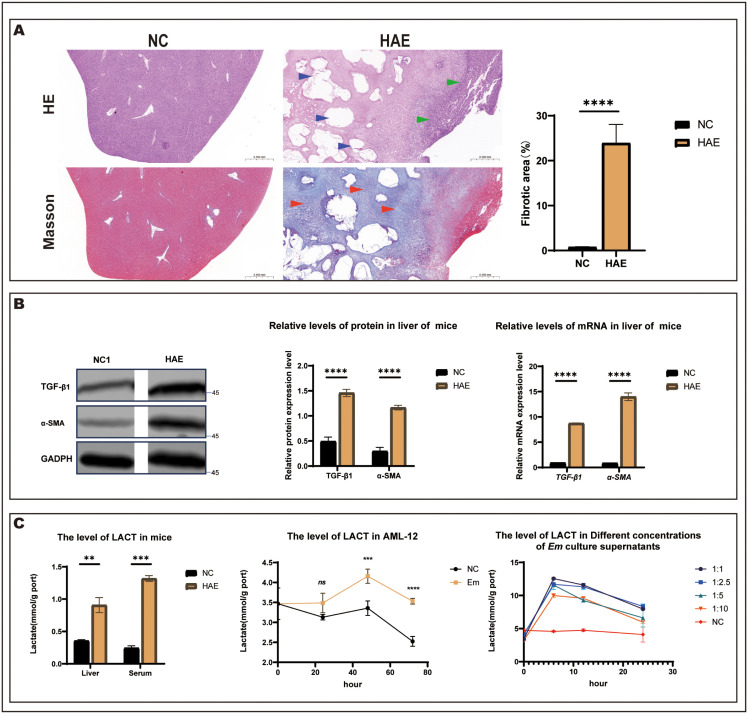
Extensive fibrosis in the liver tissue of mice with hepatic alveolar echinococcosis (HAE). A: Histopathological observations of liver tissue from control (NC) and HAE mice by hematoxylin–eosin (HE; top) and Masson’s trichrome (bottom) staining, with quantitative analysis of fibrosis area (n = 3; ****P < 0.0001, two-tailed unpaired *t*-*t*est). Blue arrows indicate vesicular structures in HAE lesions, green arrows mark inflammatory cell bands, and red arrows denote fibrotic areas. B: Western blot analysis of fibrosis-related proteins (TGF-β1 and α-SMA), relative mRNA quantification, and statistical comparison of expression levels in liver tissue from NC and HAE mice (n = 3; ****P < 0.0001, two-tailed unpaired *t*-tes*t*). C: Lactate concentrations in liver tissues and serum of NC and HAE mice, and a line graph showing time-dependent changes in lactate levels in AML-12 hepatocytes co-cultured with *E. multilocularis* protoscolex culture supernatant for 72 h (n = 3; ****P < 0.0001, two-tailed unpaired *t*-test).

*E. multilocularis* infection is well known for triggering progressive hepatic fibrosis in host tissues. To characterize these pathological changes, liver sections from infected and control mice were examined using H&E and Masson’s trichrome staining. Infected mice displayed pronounced histopathological changes, including distinct parasitic cystic structures, extensive fibrotic septa, and dense inflammatory infiltrates surrounding lesion areas (Fig 1A). Quantitative analysis showed that fibrotic regions within HAE lesions were significantly expanded compared with control liver tissues (Fig 1A). In addition, immunofluorescence and immunohistochemistry staining for Collagen I and Collagen III were performed in the livers of HAE and NC mice. The results showed markedly increased Collagen I and Collagen III staining in the livers of AE mice compared with the control group, as detected by both immunohistochemistry and immunofluorescence (S1A and S1B Fig).

To further corroborate the fibrotic response, we measured the expression levels of two key fibrosis-associated markers, TGF-β and α-SMA, by RT-qPCR and WB. Both markers were significantly upregulated in infected liver samples compared with the control group (Fig 1B), confirming a strong pro-fibrotic activation following *E. multilocularis* infection.

To investigate whether *E. multilocularis*-induced hepatic fibrosis is associated with lactate accumulation and histone lactylation, we measured lactate levels in liver tissues and serum of *E. multilocularis*-infected mice. Lactate concentrations in liver tissue and serum were substantially elevated in infected mice, reaching 0.908 mmol/g protein and 1.318 mmol/g protein, respectively, compared with 0.355 mmol/g protein and 0.244 mmol/g protein in control samples (Fig 1C). These values correspond to an average increase of 155.77% in liver lactate content and 440.16% in serum lactate levels.

To further validate whether parasite-derived factors contribute to lactate accumulation, AML-12 hepatocytes were exposed to culture supernatants from *E. multilocularis* PSCs for 72 h. Lactate content in AML-12 cells began to increase by 48 h, increasing by 16.86% (from 3.557 mmol/g protein to 4.157 mmol/g protein) (Fig 1C). These findings indicate that *E. multilocularis* infection disrupts host glucose metabolism, leading to increased hepatic and systemic lactate levels, which may contribute to the progression of fibrosis.

### HK3 upregulation is associated with hepatic lactate accumulation and elevated H3K14 and H3K18 lactylation in AE mice

**Fig 2:**
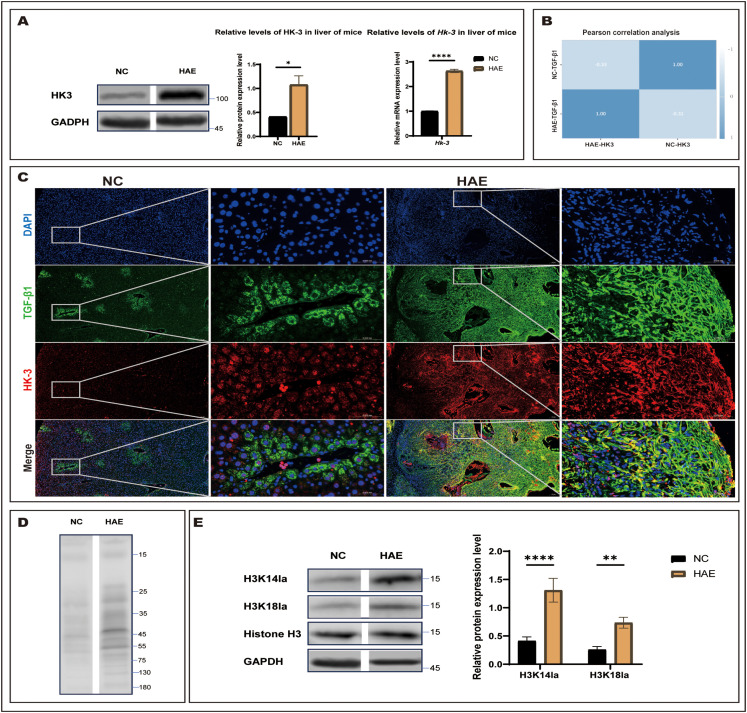
Upregulated expression of HK3 and increased histone lactylation in the liver of HAE mice. A: Quantification of HK3 protein and mRNA expression levels in hepatic alveolar echinococcosis (HAE) and control (NC) groups by WB and RT-qPCR (n = 3; ****P < 0.0001; two-tailed unpaired *t*-*t*est). B: Pearson correlation analysis between HK3 and TGF-β1 expression levels in liver tissues from HAE and NC groups. C: Immunofluorescence staining of TGF-β1 (green) and HK3 (red) in liver sections from NC and HAE mice (100×). Nuclei were counterstained with DAPI (4,’6-diamidino-2-phenylindole). D: WB analysis using a pan-lactylation antibody to assess global histone lactylation in liver tissues of NC and HAE mice. E: WB analysis of H3K18la and H3K14la expression levels in liver tissues from NC and HAE mice (n = 3; **P < 0.01; ****P < 0.0001; two-tailed unpaired *t*-test).

To investigate the factors contributing to lactate accumulation in the liver tissues of mice infected with *E. multilocularis*, we examined the expression of HKs, which catalyze the conversion of glucose into glucose-6-phosphate and initiate glycolysis. As a key rate-limiting enzyme in glycolysis, HK activity may directly affect lactate production in host tissues. We evaluated the expression levels of all four HK isoforms, HK1, HK2, HK3, and HK4. A significant increase in HK3 expression was detected in hepatic tissues of mice with HAE, whereas HK1 (*P* = 0.1075) and HK2 (*P* = 0.1537) and HK4 (*P* = 0.0062) mRNA levels showed no significant differences compared with the control group (S2A Fig). Western blot and RT-qPCR analyzes further confirmed a significant elevation of HK3 protein and mRNA expression in the livers of HAE mice relative to controls (Fig 2A). Immunofluorescence staining also demonstrated a significant expansion of HK3-positive regions within HAE mouse livers, accompanied by spatial colocalization with TGF-β expression (Fig 2B and 2C). These findings suggest that HK3 upregulation may be associated with lactate accumulation in the livers of AE mice and may be involved in the development of liver fibrosis.

Although *E. multilocularis* infection leads to HK3 upregulation and aberrant lactate accumulation in the mouse liver, it remains unclear whether this accumulation promotes histone lactylation. To address this question, WB analysis using a pan-lactylation antibody was performed on liver tissues from AE mice. Lactylation levels were significantly increased in AE samples compared with controls (Fig 2D). To identify specific histone residues undergoing lactylation in AE liver, WB analysis was conducted to assess lactylation at ten lysine sites previously reported to undergo lactyl modifications (S1C Fig). The results demonstrated a substantial elevation of lactylation in HAE mice, with H3K14la and H3K18la increasing by 216.4% and 183.7%, respectively, compared with the control group (Fig 2E). These data indicate that *E. multilocularis* infection induces pronounced histone lactylation, particularly at H3K14 and H3K18. These findings support a model in which *E. multilocularis* infection triggers HK3 upregulation in hepatic tissue, resulting in elevated lactate production. The increased lactate then increases histone lactylation, especially at H3K14 and H3K18, which may ultimately contribute to the progression of liver fibrosis.

### CUT&Tag analysis shows H3K18la lactylation in HAE mice affects TGFBR

**Fig 3:**
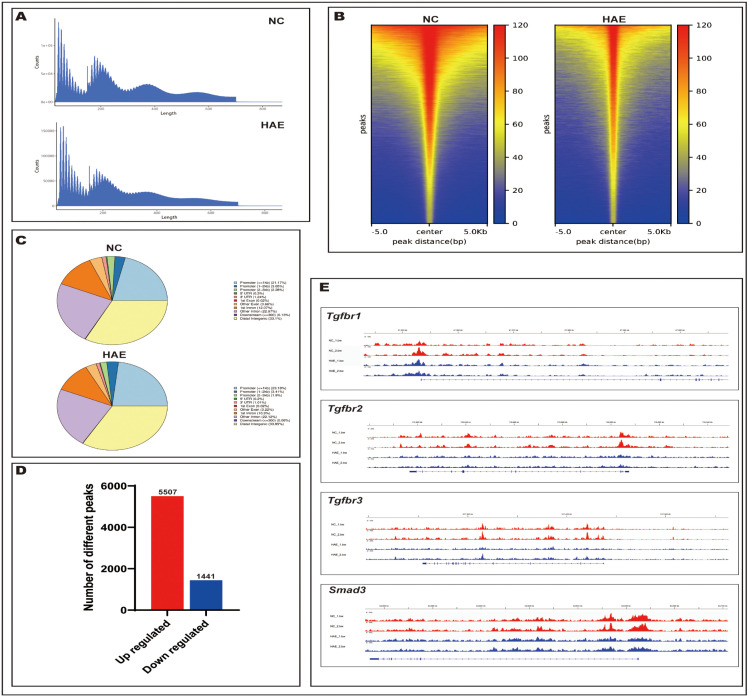
CUT&Tag analysis of liver tissues from HAE mice. **A.** Length distribution of enzymatically digested fragments in liver tissue from the control group (NC) and mice with hepatic alveolar echinococcosis (HAE). B: Distribution of sequencing reads around peak regions in liver samples from control (NC) and hepatic alveolar echinococcosis (HAE) mice. C: Functional annotation of peak-associated genomic regions. During annotation, regions were assigned in the following default priority order: promoter > 5′UTR > 3′UTR > exon > intron > downstream > intergenic, with no duplicate annotations. D: Summary of the number of differentially enriched H3K18la peaks between NC and HAE groups. E: Integrative Genomics Viewer (IGV) tracks showing H3K18la enrichment at the *Tgfbr* and *Smad3* loci in liver tissues from NC and HAE mice.

To further elucidate the functional relevance of H3K18la, particularly its role in liver fibrosis, a genome-wide CUT&Tag assay was performed on liver tissues from HAE mice using an anti-H3K18la antibody. The results showed that the overall alignment rate of sequencing reads reached approximately 90%, with fragment lengths ranging from 1 to 689 bp (Fig 3A). Using Deeptools, we mapped the distribution of H3K18la-associated reads across peak regions. Clear differences emerged between the control and HAE groups, with control samples showing a more concentrated signal around peak centers (Fig 3B).

Functional annotation of enrichment peaks using the ChIPseeker R package quantified their genomic distribution across functional regions. Compared with the control group (21.17%), the HAE group exhibited a higher proportion of peaks within promoter regions (Promoter≤1 kb, 23.18%) and fewer peaks within first intron regions (Fig 3C). Differential peak analysis identified a total of 6,948 peaks that differed between groups, including 5,507 peaks elevated and 1,441 reduced in the HAE group relative to controls (Fig 3D). Among these peaks, five regions were associated with regulation of TGF-β receptors (TGFBRs), covering all three family members, *Tgfbr1*, *Tgfbr2*, and *Tgfbr3*, along with two regions associated with *Smad3*, a downstream effector of *Tgfb1* signaling (Fig 3E). These observations indicate that H3K18la may contribute to liver fibrosis in HAE through its differently enrichment at TGFBR-related regulatory regions, facilitating activation of the TGF-β1-Smad3 axis.

### *E. multilocularis* supernatant upregulates HK3 and promotes histone lactylation and fibrosis-related protein expression in hepatocytes

**Fig 4:**
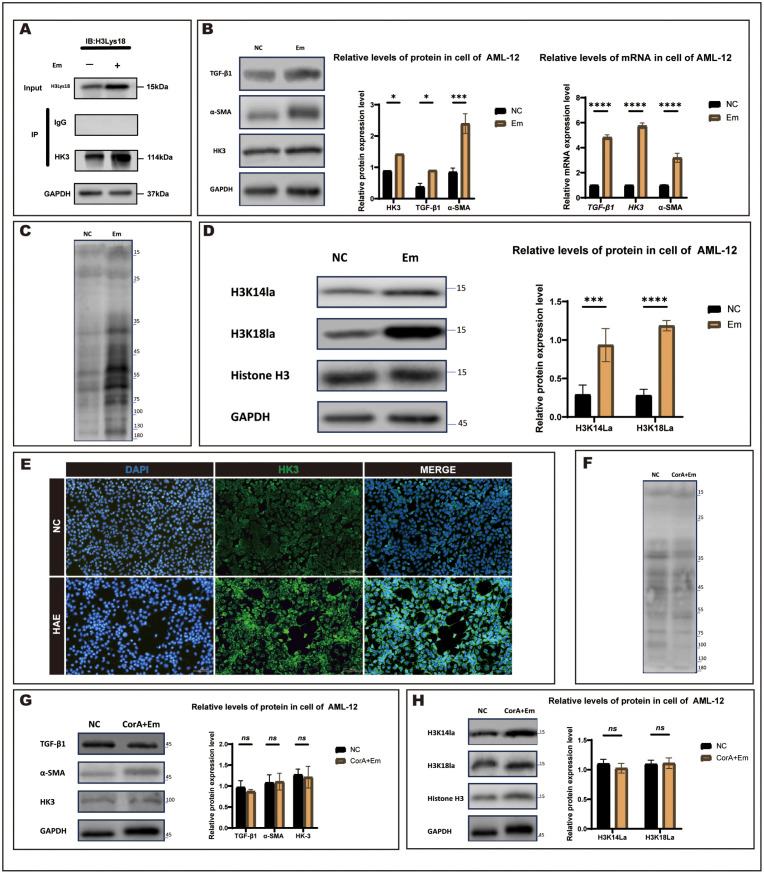
*E. multilocularis* stimulation induces histone lactylation in hepatocytes by upregulating HK3 expression. A: Co-immunoprecipitation (Co-IP) analysis of HK3 and H3K18la in *E. multilocularis*-stimulated AML-12 cell lysates. B: Western blot (WB) analysis of fibrosis-related proteins (TGF-β1 and α-SMA) and HK3, with corresponding mRNA quantification and statistical comparison of expression levels in the control (NC) and AML-12 cells stimulated with of *E. multilocularis* (Em) culture supernantant for 72 h (n = 3; *P < 0.05, ****P < 0.0001; two-way ANOVA). C: WB analysis using a pan- lactylation antibody in NC and AML-12 cells stimulated with *E. multilocularis*culture supernatant for 72 h. D: WB analysis of H3K18la and H3K14la expression in NC and stimulated AML-12 cells(72 h) (n = 3; ***P < 0.001, ****P < 0.0001*;* two-tailed unpaired *t*-test). E: Immunofluorescence staining of HK3 (green) in NC and s*t*imulated AML-12 (72 h) (magnification, 100 × ; nuclei stained with DAPI). F: WB analysis using a pan-lactylation antibody in NC and AML-12 cells treated with corosolic acid following *E. multilocularis* stimulation for 72 h (CorA + Em). G: WB analysis of TGF-β1, α-SMA, and HK3, with corresponding mRNA quantification and statistical comparison in NC and AML-12 cells treated with corosolic acid following *E. multilocularis* stimulation for 72 h (n = 3; ns = not significant; two-way ANOVA). H: WB analysis of H3K18la and H3K14la, with corresponding mRNA quantification and statistical comparison in NC and AML-12 cells treated with corosolic acid following *E. multilocularis* stimulation for 72 h (n = 3*; ns* = Non-significant; two-tailed unpaired *t*-test).

To examine the relationship between HK3 and histone lactylation, lysates from induced AML-12 cells were subjected to co-immunoprecipitation using an anti-HK3 antibody, and the immunocomplexes were probed by Western blot. H3K18la co-precipitated with HK3 (Fig 4A), indicating that HK3 is associated with H3K18-lactylated histones.

Quantitative analysis showed that, relative to controls, induced AML-12 cells exhibited significant upregulation of HK3 (by 63.36%), α-SMA (by 181.76%), and TGF-β1 (by 137.89%) (Figs 4B and S2B). Consistently, pan-lactylation signals were elevated in *E. multilocularis*-induced AML-12 cells (Fig 4C). Both H3K18la and H3K14la were significantly increased, with H3K18la showing a greater increase (by 71.27%) than H3K14la (Fig 4D). Immunofluorescence staining further confirmed increased HK3 expression in induced AML-12 cells (Fig 4E).These findings indicate that secreted factors of *E. multilocularis* drive HK3 upregulation in hepatocytes, promote histone lactylation predominantly at H3K18, and induce the expression of fibrosis-related proteins.

To assess whether corosolic acid (CorA) could suppress the *E. multilocularis*-induced fibrogenic response, AML-12 cells were pretreated with 10 μM corosolic acid for one hour before undergoing a 72-h induction with *E. multilocularis* supernatant. Pan-lactylation signals in corosolic acid-pretreated cells did not differ significantly from control levels (Fig 4F). HK3, α-SMA, and TGF-β1 levels in inhibitor-treated cells did not increase after induction and remained comparable to control levels (Fig 4G). Similarly, H3K18la levels did not differ significantly from those in the control group (Fig 4H). These findings show that CorA suppresses the *E. multilocularis-*induced increases in HK3, histone lactylatio, and fibrosis-related proteins.

### HK3 knockdown and lactate rescue reveal that HK3-derived lactate promotes H3K18 lactylation and fibrogenesis

**Fig 5:**
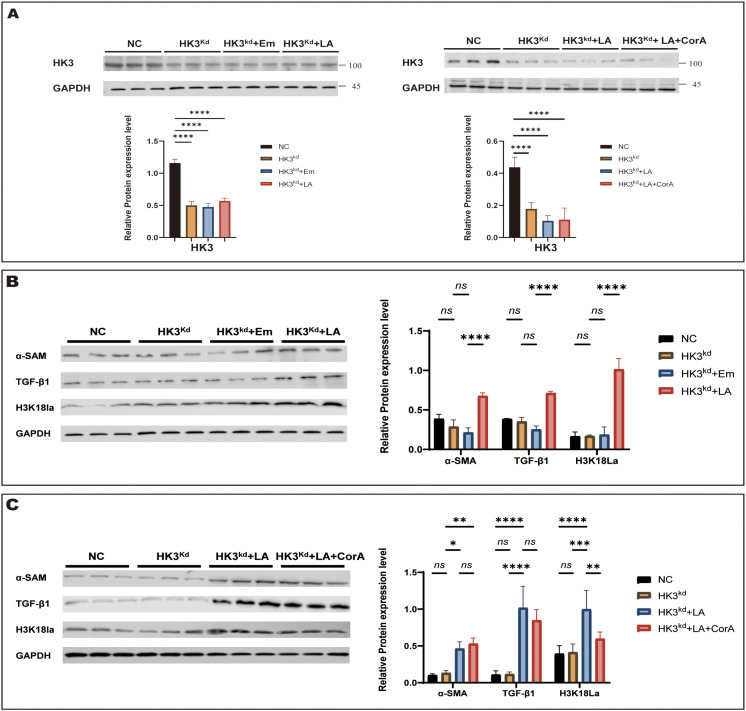
HK3-derived lactate mediates *E. multilocularis*-induced histone lactylation and fibrosis-related protein expression in AML-12 cells. A: Western blot analysis and quantification of HK3. Left panel: NC, HK3-knockdown cells (HK3^kd^), HK3^kd^ cells stimulated with *E. multilocularis* culture supernatant for 72 h (HK3^kd^+Em), and HK3^kd^ cells treated with 10 mM lactic acid for 72 h (HK3^kd^+LA) (n = 3; ****P < 0.0001; two-way ANOVA). Right panel: NC, HK3^kd^, HK3^kd^+LA, and HK3^kd^ cells treated with 10 mM lactic acid plus corosolic acid for 72 h (HK3kd+LA + CorA) (n = 3; ****P < 0.0001; two-way ANOVA). B: Western blot analysis and quantification of α-SMA, TGF-β1, H3K18la in NC, HK3^kd^, HK3^kd^+Em, and HK3^kd^+LA cells (n = 3; ****P *<* 0.0001, *ns* = not significant; two-way ANOVA). C: Western blot analysis and quantification of α-SMA, TGF-β1, and H3K18la in NC, HK3^kd^, HK3^kd^+LA, and HK3^kd^+LA + CorA cells (n = 3; *P < 0.05, **P < 0.01, ***P < 0.001, ****P *<* 0.0001, *ns* = not significant; two-way ANOVA).

To further validate the role of HK3 in lactylation-associated liver fibrosis during AE, we knocked down HK3 expression in AML-12 cells using RNAi, and the knockdown was confirmed at the protein level by WB. Following HK3 knockdown, HK3 expression remained low and was not restored by stimulation with *E. multilocularis* protoscolex culture supernatant, exogenous lactate supplementation, or corosolic acid treatment (Fig 5A), indicating that HK3 upregulation is an upstream event triggered by *E. multilocularis* stimulation rather than a secondary consequence of lactate accumulation or downstream signaling.

Following HK3 knockdown, protoscolex culture supernatant no longer induced a significant increase in H3K18la, and the upregulation of α-SMA and TGF-β1 was significantly suppressed(Fig 5B), indicating that HK3 is required for *E. multilocularis* -induced histone lactylation and fibrogenic signaling. To determine whether lactate is the key intermediate mediating these effects, we supplemented HK3-knockdown cells with 10 mM exogenous sodium lactate in the absence of protoscolex stimulation. Exogenous lactate significantly increased the levels of H3K18la, α-SMA and TGF-β1 despite HK3 knockdown (Fig 5B), demonstrating that lactate functions as the critical intermediate linking HK3 activity to histone lactylation and fibrogenesis.

We next examined whether corosolic acid could reverse this lactate-driven fibrogenic response. When corosolic acid was added to HK3-knockdown cells supplemented with exogenous lactate, it partially reduced H3K18la levels but did not suppress the lactate-induced upregulation of α-SMA and TGF-β1 (Fig 5C). Notably, since HK3 was already silenced and lactate was supplied exogenously, the residual reduction of H3K18la by corosolic acid suggests that its effect on histone lactylation is not entirely dependent on HK3-mediated lactate production.

### Corosolic acid attenuates hepatic fibrosis in *E. multilocularis*-infected mice

**Fig 6:**
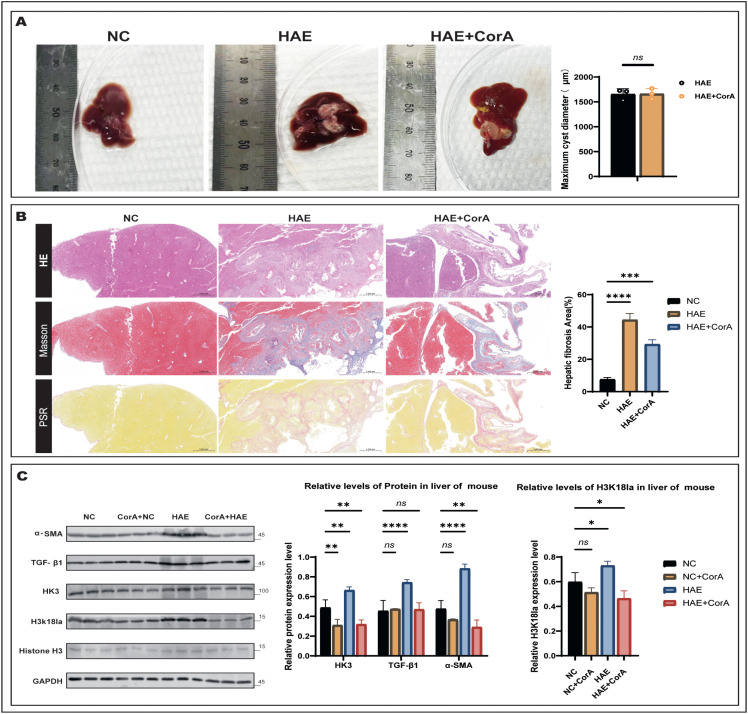
Corosolic acid treatment attenuates liver fibrosis in *E. multilocularis*-infected mice. A: Gross morphology of livers and statistical analysis of maximum cyst diameter in control mice (NC), hepatic alveolar echinococcosis (HAE) mice, and HAE mice treated with corosolic acid (HAE + CorA) (n = 3; *ns* = not significant; two-tailed unpaired *t*-test). B: Histopathological staining of liver tissues using hematoxylin–eosin (HE; top), Masson’s trichrome (middle), and Sirius Red (bottom) from NC, HAE, and HAE + CorA groups, with comparative quantification of fibrotic area ratios (n = 3; ***P < 0.001, ****P *<* 0.0001; two-way ANOVA). C: Western blot analysis and quantitative comparison of α-SMA, TGF-β1, HK3, H3K18la, and H3K14la in liver tissues from NC, HAE, and HAE + CorA groups (n = 3; *P < 0.05, **P < 0.01, ****P < 0.0001*, ns* = not significant*;* two-way ANOVA).

To explore whether the HK3–lactylation–TGF-β1 axis can be targeted during hepatic fibrosis *in vivo*, *E. multilocularis*-infected mice were treated with corosolic acid (10 mg/kg, intraperitoneally, every 3 days for a total of 5 doses). Although corosolic acid did not significantly change maximum cyst diameter (Fig 6A), histopathology showed a clear reduction in hepatic fibrosis area in the drug-treated group compared with controls (Fig 6B). Molecular analysis revealed that HK3 levels in corosolic acid-treated mice showed a significantly reduced after *E. multilocularis* infection, rather than the increase observed in untreated AE mice (Figs 6C and S2C). TGF-β1 expression remained stable without the increase typically seen during infection, and α-SMA expression showed a significant reduction in the liver tissues of corosolic acid-treated AE mice (Figs 6C and S2C). Furthermore, H3K18la levels were significantly decreased in corosolic acid-treated mice, indicating that corosolic acid prevented the *E. multilocularis* infection-induced increase in histone lactylation (Fig 6C). These findings suggest corosolic acid alleviates *E. multilocularis*–induced hepatic fibrosis in association with reduced HK3 expression and histone lactylation, particularly during early infection. The results also suggest that targeting HK3 may offer a potential strategy for slowing fibrosis progression in AE.

## Discussion

Hepatic fibrosis, a key pathological feature of HAE, remains a major challenge in disease management. It has traditionally been regarded as a process mainly driven by inflammation and extracellular matrix deposition. In this study, however, an additional regulatory layer involving histone lactylation was identified. *E. multilocularis* infection led to a significant upregulation of HK3 expression, which contributed to lactate accumulation in hepatic tissues. This increase in lactate was accompanied by activation of H3K14 and H3K18 lactylation, promoting TGF-β1 expression and hepatic fibrosis progression. Pathogen-infected cells often undergo metabolic reprogramming, including increased glycolysis resembling the Warburg effect, to meet their energy and biosynthetic precursor demands [12,30,31]. Such metabolic changes are commonly achieved through upregulated expression of glucose transporters and hexokinases. *E. multilocularis* infection is known to induce broad activation of HSCs and stimulate a strong immune response in the liver [32–34], which may elevate metabolic activity in HAE liver tissues [35]. These metabolic changes can upregulate hexokinase expression, contributing to lactate accumulation. These results suggest that HK3 plays an important role in this process and may be a significant contributor to the development of HAE-associated fibrosis.

*E. multilocularis* infection can alter the expression of multiple host genes. Previous studies have shown that the expression levels of miR-106b-25 and miR-93 are elevated in mice with HAE, where they contribute to the liver damage caused by *E. multilocularis* [36]. miR-106b-25 regulates the expression of TGF-β, which may be associated with the development of fibrosis in HAE [37,38]. In activated HSCs, HK expression and activity increase, accelerating glucose metabolism and leading to elevated lactate production [14,39,40]. This increase in lactate is a direct result of increased glycolysis. During fibrotic progression, quiescent HSCs become activated and differentiate into myofibroblasts, which serve as the main collagen-producing cells in the liver. Research has demonstrated that HK2 plays a key role in HSC activation and the pathogenesis of hepatic fibrosis. HK2 participates in glycolysis by catalyzing the key phosphorylation step in glucose metabolism, increasing lactate production. This lactate is not only a metabolic by-product but also regulates gene expression through histone lactylation at H3K18la, promoting HSC activation [14]. Compared with HK2, research on HK3 in hepatic fibrosis remains limited. However, as another hexokinase family member, HK3 may also participate in hepatic metabolic reprogramming and HSC activation. In this study, HK3 expression was significantly elevated in the livers of *E. multilocularis*-infected mice, indicating that glucose metabolic reprogramming in HAE may have unique characteristics. Moreover, elevated HK3 expression and activity promote lactate production and accumulation, and by increasing histone lactylation (H3K14la and H3K18la) in HAE liver tissues, they also promote the expression of fibrosis-associated factors such as TGF-β1 and α-SMA. These findings indicate that *E. multilocularis* infection elicits a sequential cascade: upregulated HK3 expression and enhanced enzymatic activity increase lactate accumulation, which, in turn, drives histone lactylation (H3K14la and H3K18la) in HAE liver tissues. This epigenetic reprogramming activates transcription of pro-fibrotic factors, including TGF-β1 and α-SMA, defining a key mechanistic pathway by which the parasite promotes liver fibrosis *via* the HK3–histone lactylation–fibrotic factor axis.

The subsequent findings showed that the accumulated lactate in AE mice served as a substrate for histone epigenetic modifications. These modifications affected the expression of fibrosis-related genes, including *TGF-β1* and *α-SMA*, thereby affecting the overall progression of AE. Histone lactylation is known to contribute to HSC activation and hepatic fibrosis, and has recently become a focal point in studies on liver disease mechanisms and therapeutic targets [14,41–43]. In this study, H3K18 and H3K14 lactylation levels were significantly elevated in AE mouse livers. The increase in H3K18la correlated with HSC activation and the subsequent deposition of TGF-β1 and α-SMA, which are hallmarks of hepatic fibrosis. Chromatin profiling using CUT&Tag further revealed that H3K18la was differentially enriched at the regulatory regions of TGFBR, suggesting that lactylation may contribute to hepatic fibrosis by regulating TGFBR signaling. Furthermore, *in vitro* experiments with corosolic acid demonstrated that inhibition of HK3 activity reduced lactate levels, lowered histone lactylation, and decreased the expression of fibrosis-related proteins. This intervention also slowed or partially reversed fibrosis progression in AE mice. These results indicate that H3K18 lactylation is a key molecular event in AE-associated hepatic fibrosis. This work provides new insight into the underlying pathological mechanisms and suggests that inhibitors targeting H3K18 lactylation may hold therapeutic potential.

To determine whether HK3 is functionally required for these changes, we performed loss-of-function and rescue experiments *in vitro*. Silencing HK3 in AML-12 cells significantly suppressed the *E. multilocularis*-induced increase in H3K18la and the upregulation of α-SMA and TGF-β1, indicating that HK3 is required for the induction of histone lactylation and fibrogenic signaling. Importantly, HK3 expression itself was not altered by exogenous lactate or corosolic acid, placing HK3 upregulation upstream as an event triggered by *E. multilocularis* stimulation rather than a downstream consequence of lactate accumulation. When exogenous lactate was supplied to HK3-silenced cells, H3K18la and fibrosis-associated proteins were significantly increased despite the absence of HK3 activity, demonstrating that lactate is the critical intermediate linking HK3 to histone lactylation and fibrogenesis.

Our co-immunoprecipitation experiments showed that H3K18la co-precipitated with HK3 in *E. multilocularis*-stimulated hepatocytes. However, HK3 is a glycolytic enzyme predominantly localized to the cytoplasm [44]. We therefore do not interpret this co-precipitation as evidence of a direct interaction between HK3 and nuclear histones. Instead, we propose an indirect mechanism, in which cytoplasmic HK3 enhances lactate production, and lactate, which can enter the nucleus, serves as the substrate for H3K18 lactylation. This interpretation is consistent with our lactate-rescue experiment. Nuclear–cytoplasmic fractionation and orthogonal localization assays in future studies would help define the nature of this association.

Although corosolic acid attenuated the infection- and stimulation-induced increases in HK3 expression, histone lactylation, and fibrosis markers, it retained a partial ability to lower H3K18la even when HK3 was silenced and lactate was supplied exogenously. This residual activity indicates that corosolic acid is not a strictly HK3-specific agent, consistent with the broad pharmacological profile of pentacyclic triterpenoids, which also exert anti-inflammatory, antioxidant, and apoptosis-regulating effects [45–48]. Notably, once lactate had accumulated, corosolic acid could no longer reverse the fibrogenic response, suggesting that its anti-fibrotic effect operates primarily upstream by limiting HK3-driven lactate production rather than by directly targeting the downstream fibrotic program. Therefore, corosolic acid is best regarded as an upstream metabolic intervention associated with this pathway rather than direct proof of an HK3-specific mechanism. While our *in vitro* knockdown experiments establish the functional requirement of HK3, an *in vivo* HK3 conditional knockout model is still needed to confirm its specific contribution to lactylation and fibrosis in HAE, which represents an important direction for future work.

Previous studies on lactylation in hepatic fibrosis, cancer, and chemically models have mostly focused on the role of HK2 [14,49,50]. However, why HK3, rather than HK2, plays a predominant role in HAE-associated lactylation remains unclear, and whether this reflects a distinct metabolic feature unique to parasitic infection also warrants further investigation. This study identified two major lactylation sites, H3K14 and H3K18, *in vivo* and *in vitro*. Although CUT&Tag analysis revealed differential enrichment of H3K18la at TGFBR regulatory regions, the involvement of H3K14la in fibrosis remains unknown. Future studies will determine whether H3K14la participates in tissue fibrosis and whether H3K14la and H3K18la act synergistically to promote liver fibrotic progression. Finally, as AML-12 is a hepatocyte line rather than a hepatic stellate cell, it does not itself undergo fibrosis but acts as an upstream cell that responds to injury and secretes pro-fibrotic factors such as TGF-β1 [51,52]. How this upstream hepatocyte response translates into matrix remodeling by stellate cells remains to be defined and warrants further study using hepatic stellate cell and co-culture models.

In conclusion, this study identified extensive histone lactylation in liver lesions caused by *E. multilocularis* infection and provided functional evidence for a causal link between this modification and host hepatic fibrosis in AE. Specifically, infection with *E. multilocularis* protoscoleces induced HK3 upregulation in the host liver, which altered hepatic glucose metabolism and led to lactate accumulation. This pronounced lactate accumulation increased histone lactylation, particularly at H3K18, which was associated with the transcriptional activation of fibrosis-related genes including TGF-β1 and α-SMA. Loss-of-function and lactate-rescue experiments confirmed that HK3-driven lactate production acts upstream of histone lactylation and fibrogenesis, while corosolic acid attenuated this process primarily by limiting HK3-driven lactate production. Together, these findings highlight the central role of HK3-mediated histone lactylation in the pathogenesis of HAE, and suggest that targeting HK3 may offer a promising therapeutic strategy for this condition.

## Supporting information

S1 Table The information on the primers in qPCR. (DOCX)

S2 Table Antibody information. (DOCX)

S1 Fig Immunohistochemical and immunofluorescence staining of fibrosis marker proteins and histone lactylation in liver tissues of hepatic alveolar echinococcosis (HAE) and control mice. A: Collagen I (COL I; top) and Collagen III (COL III; bottom) immunohistochemical staining in liver tissues from control (NC) and HAE mice (100×). B: Immunofluorescence staining of Collagen I (orange) and Collagen III (purple) in liver sections from NC and HAE mice, nuclei were counterstained with DAPI (4,’6-diamidino-2-phenylindole) (100×). C: Relative levels of histone lactylation at specific lysine sites in liver tissues from NC and HAE mice (n = 4; ns = not significant; two-tailed unpaired t-test).
(TIF)

S2 Fig Expression of hexokinase isoforms and fibrosis-related markers in hepatic alveolar echinococcosis (HAE). A: Relative mRNA expression of hexokinase isoforms in mouse liver tissue (n = 4; ***P < 0.001*, ns =* not significant; two-way ANOVA). B: Relative mRNA expression levels of HK3 and fibrosis-related proteins in AML-12 cells infected with *Echinococcus multilocularis* (Em) with or without corosolic acid intervention (Em + CorA), compared with NC (n = 4; ***P < 0.0001; two-way ANOVA). C: Relative mRNA expression of HK3 and fibrosis-related proteins in liver tissues from NC, corosolic acid-treated (CorA), HAE, and HAE + CorA groups (n = 4; **P < 0.01, ****P < 0.0001*, ns =* not significant; two-way ANOVA).
(TIF)

S1 Raw Gel Full-length uncropped Western blot images. (DOCX)

## Abbreviation:

AE: alveolar echinococcosis;
*E. multilocularis*: *Echinococcus multilocularis*
HK3: hexokinase-3
HK2: Hexokinase-2
HK1: hexokinase-1
HK4: hexokinase-4
WB: western blot;
H3K18la: histone H3 lysine 18 lactylation
DALYs: disability-adjusted life years;
TGF-β1: transforming growth factor β1
Acta2: alpha-smooth muscle actin
RT-qPCR: reverse transcription quantitative PCR
FGF: fibroblast growth factor;
iNOS: inducible nitric oxide synthase;
NF-κB: nuclear factor κB
IL-10: interleukin-10
HSCs: hepatic stellate cells;
HAE: hepatic alveolar echinococcosis.
