# Supplementary material for: Hexokinase-3 upregulation and histone H3K18 lactylation mediate murine liver fibrosis induced by Echinococcus multilocularis
Source: PLoS Negl Trop Dis. 2026 Jul 31;20(7):e0014582. doi: 10.1371/journal.pntd.0014582 (PMC13460742; doi:10.1371/journal.pntd.0014582)
Supplement: Supplementary file 1 [file pntd.0014582.s001.docx]

**S1 Table. The information on the primers in qPCR**

| **Gene Name** | **Species** | **Forward Primer (5′→3′)** | **Reverse Primer (5′→3′)** |
| --- | --- | --- | --- |
| *GAPDH* | mouse | GCCTCCTCCAATTCAACCCT | CCCAATACGGCCAAATCCGT |
| *TGF-β* | mouse | GCTGAACCAAGGAGACGGAATA | GGTTCATGTCATGGATGGTGC |
| *α-SMA* | mouse | AGCCATCTTTCATTGGGATGG | CCCCTGACAGGACGTTGTTA |
| *HK1* | mouse | CGAGCTGAAGGATGACCAAGT | TTTTCTGAGCCGTCCGGAAT |
| *HK2* | mouse | ATCGCCGGATTGGAACAGAA | CTGTCCGACCGCTTTGAAAC |
| *HK3* | mouse | AATATGTGTGCGTGGCTGTG | ACAGCCACTTGCAATGTCTG |
| *HK4* | mouse | AGCTGGTGGAGAAGGTGATG | GGTGTCGATGGTGATGGTAG |
