# Supplementary material for: Hexokinase-3 upregulation and histone H3K18 lactylation mediate murine liver fibrosis induced by Echinococcus multilocularis
Source: PLoS Negl Trop Dis. 2026 Jul 31;20(7):e0014582. doi: 10.1371/journal.pntd.0014582 (PMC13460742; doi:10.1371/journal.pntd.0014582)
Supplement: Supplementary file 2 [file pntd.0014582.s002.docx]

**S2 Table. Antibody information**

| **Protein name** | **Commodity manufacturer** | **Item number** | **Dilution ratio for WB** | **Dilution ratio for IF** |
| --- | --- | --- | --- | --- |
| Gadph | Abcam | ab9485 | 1：2000 |  |
| α-SMA | Abcam | ab5694 | 1：2000 |  |
| TGF-β1 | Jingjie | PTM-6897 | 1：1000 | 1：200 |
| HK3 | Abclonal | A8428 | 1：2000 | 1：200 |
| Anti-L-Lactyl Lysine Rabbit mAb | Jingjie | PTM-1401RM | 1：1000 |  |
| Anti-L-Lactyl-Histone H3 (Lys14) Rabbit mAb | Jingjie | PTM-1414RM | 1：1000 |  |
| Anti-L-Lactyl-Histone H3 (Lys18) Rabbit mAb | Jingjie | PTM-1406RM | 1：1000 |  |
| Anti-L-Lactyl-Histone H2B (Lys16) Rabbit mAb | Jingjie | PTM-1424RM | 1：1000 |  |
| Anti-L-Lactyl-Histone H3 (Lys9) Rabbit mAb | Jingjie | PTM-1419RM | 1：1000 |  |
| Anti-L-Lactyl-Histone H4 (Lys5) Rabbit mAb | Jingjie | PTM-1407RM | 1：1000 |  |
| Anti-L-Lactyl-Histone H4 (Lys8) Rabbit mAb | Jingjie | PTM-1415RM | 1：1000 |  |
| Anti-L-Lactyl-Histone H4 (Lys12) Rabbit mAb | Jingjie | PTM-1411RM | 1：1000 |  |
| Anti-L-Lactyl-Histone H4 (Lys16) Rabbit mAb | Jingjie | PTM-1417RM | 1：1000 |  |
| Goat Anti-Rabbit IgG H&L | Abcam | ab6721 | 1：10000 | |
| Goat anti-Rabbit IgG (H+L) Highly Cross-Adsorbed Secondary Antibody | Thermo Fisher Scientific | A32731 |  | 1：500 |
