# Supplementary material for: Hexokinase-3 upregulation and histone H3K18 lactylation mediate murine liver fibrosis induced by Echinococcus multilocularis
Source: PLoS Negl Trop Dis. 2026 Jul 31;20(7):e0014582. doi: 10.1371/journal.pntd.0014582 (PMC13460742; doi:10.1371/journal.pntd.0014582)
Supplement: Supplementary file 3 [file pntd.0014582.s005.docx]

Figure 1 B

TGF-β1

α-SMA

GAPDH

Figure 2 A

HK3

GAPDH

Figure 2 D

Pan-kla NC is second HAE is fifth

Figure 2 E

H3K14La

H3K18La

Histone H3

GAPDH

Figure 4 A

IP HK3

Input H3K18La

GAPDH

Figure 4 B

TGF-β1

α-SMA

HK3

GAPDH

Figure 4 C

Pan-kla

Figure 4 D

H3K14La

H3K18La

Histone H3

GAPDH

Figure 4 F

Pan-kla

Figure 4 G

TGF-β1

α-SMA

HK3

GAPDH

Figure 4 H

H3K14La

H3K18la

Histone H3

GAPDH

Figure 5 A

Left HK3

Left GAPDH

Right HK3

Right GAPDH

Figure 5 B

α-SMA

TGF-β1

H3K18La

GAPDH

Figure 5 C

α-SMA

TGF-β1

H3K18La

GAPDH

Figure 6 C

α-SMA

TGF-β1

HK3

H3K18La

Histone H3

GAPDH

S1 Figure C

H3K9me3

H4K5cr

H4K8ac

H4K16ac

H2BK16La

H4K12me1
